# Biochar-Based Graphitic Carbon Nitride Adorned with Ionic Liquid Containing Acidic Polymer: A Versatile, Non-Metallic Catalyst for Acid Catalyzed Reaction

**DOI:** 10.3390/molecules25245958

**Published:** 2020-12-16

**Authors:** Samahe Sadjadi, Fatemeh Koohestani, Majid Heravi

**Affiliations:** 1 Gas Conversion Department, Faculty of Petrochemicals, Iran Polymer and Petrochemicals Institute, P.O. Box 14975112, Tehran 1497713115, Iran; f.koohestani10@gmail.com; 2 Department of Chemistry, School of Science, Alzahra University, P.O. Box 1993891176, Vanak, Tehran 1993891176, Iran

**Keywords:** ionic liquid, catalyst, biochar, graphitic carbon nitride, Knoevenagel condensation, dihydropyrimidinones

## Abstract

A novel biochar-based graphitic carbon nitride was prepared through calcination of *Zinnia grandiflora* petals and urea. To provide acidic and ionic-liquid functionalities on the prepared carbon, the resultant biochar-based graphitic carbon nitride was vinyl functionalized and polymerized with 2-acrylamido-2-methyl-1-propanesulfonic acid, acrylic acid and the as-prepared 1-vinyl-3-butylimidazolium chloride. The final catalytic system that benefits from both acidic (–COOH and –SO_3_H) and ionic-liquid functionalities was applied as a versatile, metal-free catalyst for promoting some model acid catalyzed reactions such as Knoevenagel condensation and Biginelli reaction in aqueous media under a very mild reaction condition. The results confirmed high activity of the catalyst. Broad substrate scope and recyclability and stability of the catalyst were other merits of the developed protocols. Comparative experiments also indicated that both acidic and ionic-liquid functionalities on the catalyst participated in the catalysis.

## 1. Introduction

Ionic liquids, ILs, are an interesting class of salts that are formed from organic cations and organic or inorganic anions [1,2,3]. A vast range of heterocycles such as imidazoles and pyridines can be utilized as organic cations in ILs. Similarly, various organic and inorganic anions can be incorporated in the structure of ILs to prepare task specific ILs [1,4,5]. The importance of ILs is due to their outstanding properties, such as high electric conductivity, low cost and thermal stability [6,7,8]. These features ascertained broad utility of ILs in various research sectors, including catalysis. ILs are considered as perfect, metal-free catalysts that can meet the principles of green chemistry. Mostly, ILs have been heterogenized by using conventional supporting materials.

Graphitic carbon nitride (GCN) is a metal-free, semiconductor carbon nanomaterial, containing carbon and nitrogen that can be easily prepared from cheap starting materials under calcination condition [9,10,11,12]. Among various precursors, urea is utilized frequently due to its appealing properties, such as low cost, nontoxicity and abundance [13]. Since its discovery, GCNs have found vast applications in various research domains, such as catalysis, photocatalysis, fuel cells and separation [14,15,16,17]. Regarding the catalysis, GCNs can be employed both as catalysts and catalyst supports [18].

Biochars (BCs) are low-cost and readily available carbon materials that can be derived from biosources through thermal treatment, such as pyrolysis, gasification and hydrothermal carbonization [19,20,21,22,23,24]. Biochars are considered as promising candidates for replacing expensive carbon materials for supporting catalytic species. However, they suffer from some drawbacks, such as a lack of functional groups and low textural properties [25,26,27,28,29]. To handle these issues, and improve the features of biochars, they are mostly treated chemically or physically.

Knoevenagel condensation is one of the well-established chemical transformations that leads to the formation of α,β-unsaturated chemicals [30,31]. This is a fundamental reaction that is extensively used for the synthesis of drugs, functional polymers and many important fine chemicals, such as dyes, herbicides, fragrances and optoelectronic materials. This reaction is usually catalyzed by basic [32,33,34] or acidic catalysts [35,36,37]. To promote this reaction, various catalysts, including metallic and non-metallic catalysts can be utilized [38,39,40].

The conventional reaction, used for the synthesis of dihydropyrimidinones (DHPMs) is the Biginelli reaction. This reaction has disadvantages, such as harsh reaction conditions, prolonged time, poor yields due to the formation of side products, and use of various volatile organic solvents. Hence, many catalytic systems have been improved for the synthesis of DHPMs, including magnetic catalysts [41,42,43], ionic liquids [44,45,46], carbohydrate [47,48], Bronsted acids [49,50,51] and Lewis acid [52,53,54]. On the other hand, the importance of this reaction lies in the biological properties of DHPMs and wide utility for pharmaceutical purposes, such as antiviral, antimalarial, antihypertensive, antifungal, antitubercular and antibacterial properties [55,56,57]. This multicomponent reaction is mostly promoted by an appropriate catalyst [58,59,60,61].

We are interested in developing heterogeneous catalysts with the use of natural raw materials [62,63,64,65]. In this line, we have recently reported non-metallic catalysts that were designed by introduction of ILs or acidic moieties on a natural supporting material [8,66]. In the continuation of our studies, we wish to report a versatile catalyst, BC@GCN-P-IL, that benefits from both acidic and IL moieties.

## 2. Results and Discussion

**Figure 1:**
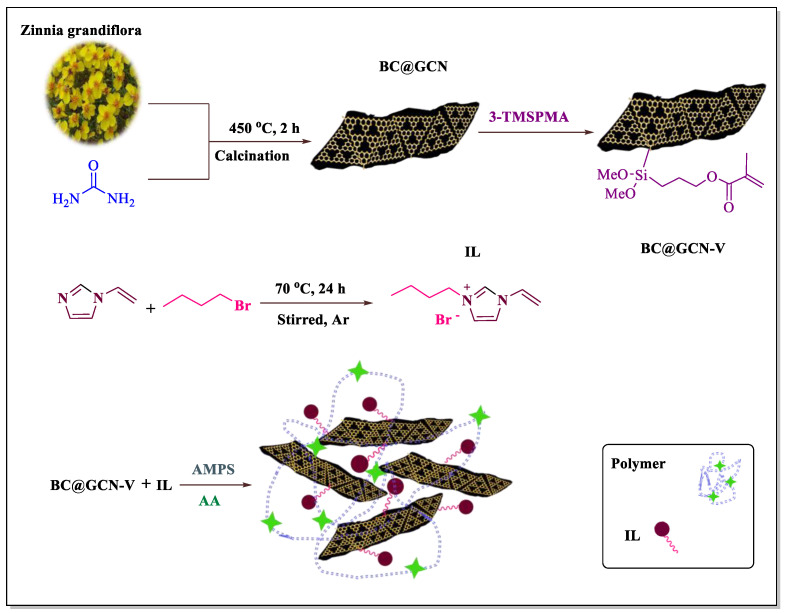
Schematic illustration of preparation of BC@GCN-P-IL.

To prepare the catalyst, *Zinnia grandiflora* petals were mixed with urea and calcined to furnish biochar-based graphitic carbon nitride (BC@GCN). The latter, was then vinyl functionalized with 3-(trimethoxysilane) propyl methacrylate (3-TMSPMA) to furnish BC@GCN-V and subsequently tolerated polymerization reaction with 1-vinylimidazole, 2-acrylamido-2-methyl-1-propanesulfonic acid (AMPS) and acrylic acid (AA), Figure 1. The polymeric moiety (P-IL) that has been grown on BC@GCN contained both acidic (–COOH and –SO_3_H) [66] and IL moieties on its backbone. Hence, it was assumed that it could promote various classic, acid-catalyzed chemical transformations efficiently under mild reaction condition. To verify this issue, the activity of the catalyst for the Knoevenagel condensation reaction and Biginelli reaction was examined. Moreover, the catalyst recyclability and the stability of the reused catalyst were studied. On the other hand, in a comparative study, the contribution of the hybrid components to the catalysis was appraised.

### 2.1. Validation of Formation of BC@GCN-P-IL

**Figure 2:**
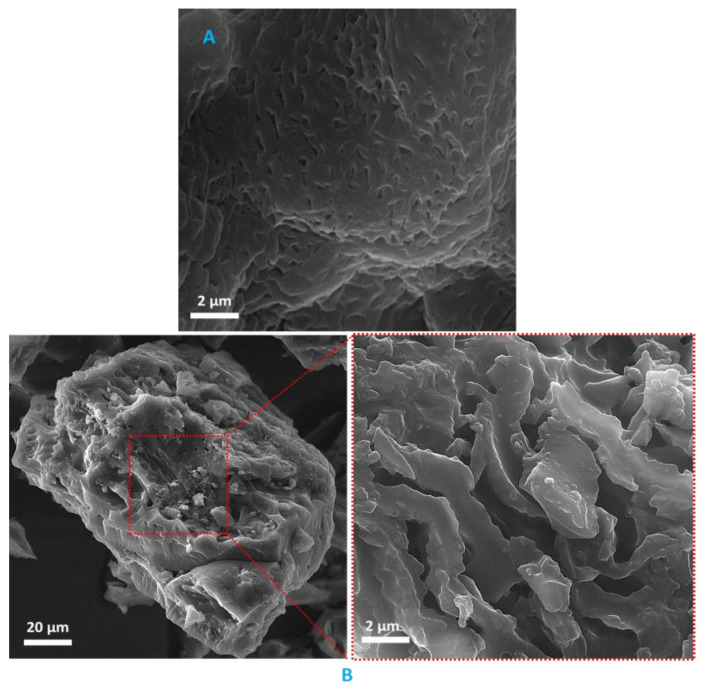
(**A**) FE-SEM image of BC@GCN and (**B**) FE-SEM images of BC@GCN-P-IL.

In Figure 2, the FE-SEM images of BC@GCN-P-IL are presented. As depicted, the catalyst exhibited aggregated-like morphology. Aggregation can be induced by the non-covalent interactions, such as hydrogen bonds and electrostatic interactions between the functionalities on BC@GCN-P-IL. Comparison of the images of the catalyst with that of BC@GCN, Figure 2, indicated that the two samples exhibited distinguished morphologies. More precisely, introduction of the polymeric moiety on BC@GCN changed its morphology.

**Figure 3:**
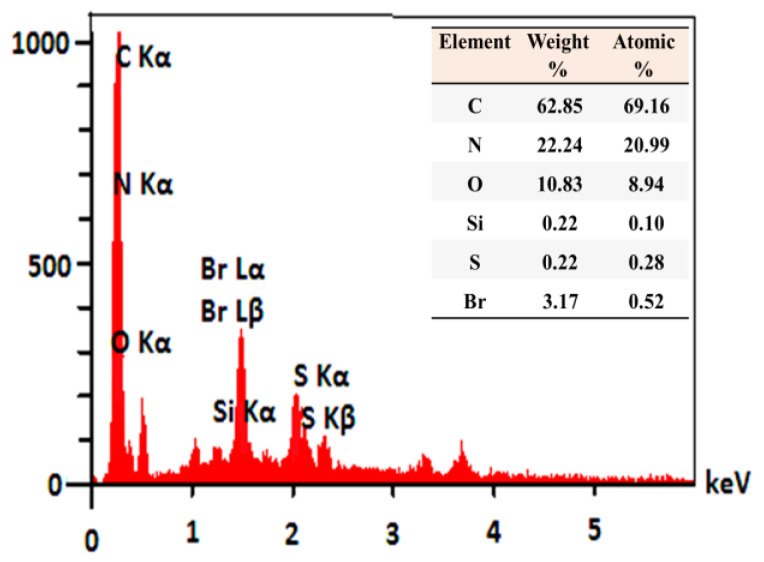
EDS analysis of BC@GCN-P-IL.

Energy dispersive spectroscopy (EDS) analysis was also performed to further analyze BC@GCN-P-IL. As shown in Figure 3, C, N, O, Br, Si and S atoms were detected in EDS analysis. C, N and O atoms are representative of BC@GCN. Notably, C and O atoms are also indicative of AA. The presence of Br, along with C and N atoms, on the other hand, can be ascribed to the P-IL moiety.

**Figure 4:**
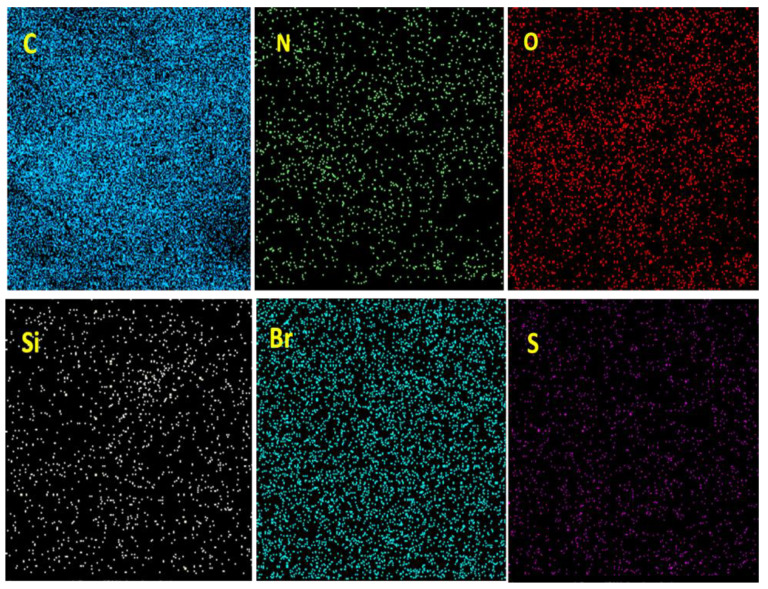
Elemental mapping analysis of BC@GCN-P-IL.

Mapping analysis of BC@GCN-P-IL is depicted in Figure 4. As illustrated, S atoms were almost uniformly dispersed. N, C and Br atoms were also dispersed homogeneously, approving the uniform dispersion of IL on BC@GCN.

**Figure 5:**
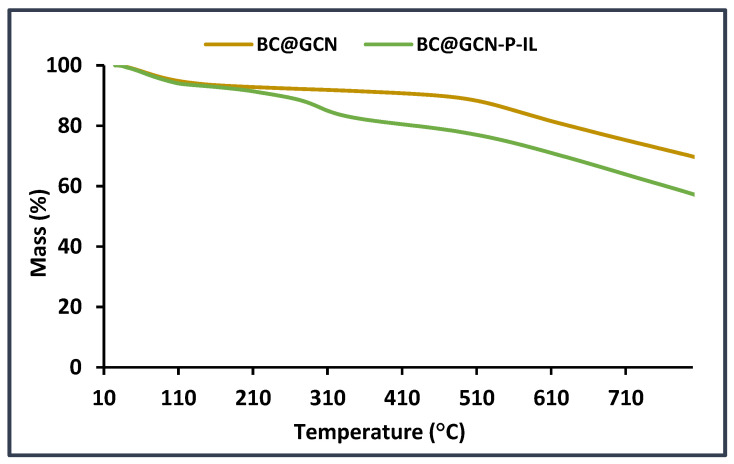
Thermograms of BC@GCN and BC@GCN-P-IL.

To further approve the conjugation of P-IL on the backbone of the catalyst, TG curves of BC@GCN and BC@GCN-P-IL were recorded, Figure 5. Comparison of the two recorded curves demonstrated that the thermal stability of BC@GCN-P-IL was inferior compared to BC@GCN. In fact, BC@GCN exhibited only two weight losses at 120 °C and 510 °C [67]. In the TG curve of BC@GCN-P-IL, an additional weight loss at the range of 240–320 °C (10 wt %) could be detected that corresponded to the degradation of P-IL.

**Figure 6:**
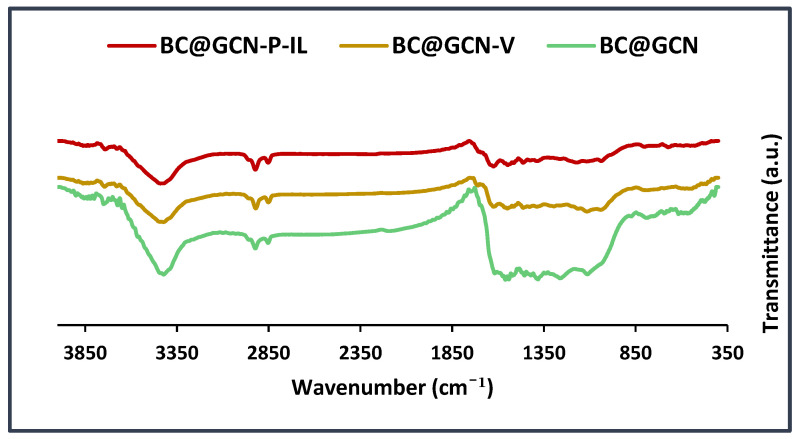
FTIR spectra of BC@GCN, BC@GCN-V and BC@GCN-P-IL.

The next characterization technique was FTIR spectroscopy. To verify the formation of each intermediate, the FTIR spectra of BC@GCN, BC@GCN-V and BC@GCN-P-IL were recorded and compared, Figure 6. As illustrated, the FTIR spectrum of BC@GCN showed the absorbance bands at 3418 cm^−1^ (–OH functionality), 2920 cm^−1^ (–CH_2_ functionality), 1623 cm^−1^ (–C=C functionality), 1033 cm^−1^ and 1409 cm^−1^ (–C-O functionality). The FTIR spectrum of BC@GCN-V contained the absorbance bands observed in BC@GCN. Moreover, an additional band at 1723 cm^−1^ can be discerned in this spectrum that is indicative of conjugation of 3-TMSPMA. FTIR spectrum of BC@GCN-P-IL also exhibited the bands of BC@GCN and an additional band at 1700 cm^−1^ that is indicative of the carbonyl functionality in polymeric moiety. Notably, the main characteristic band of IL moiety, i.e., –C=N band overlapped with the bands of BC@GCN.

**Figure 7:**
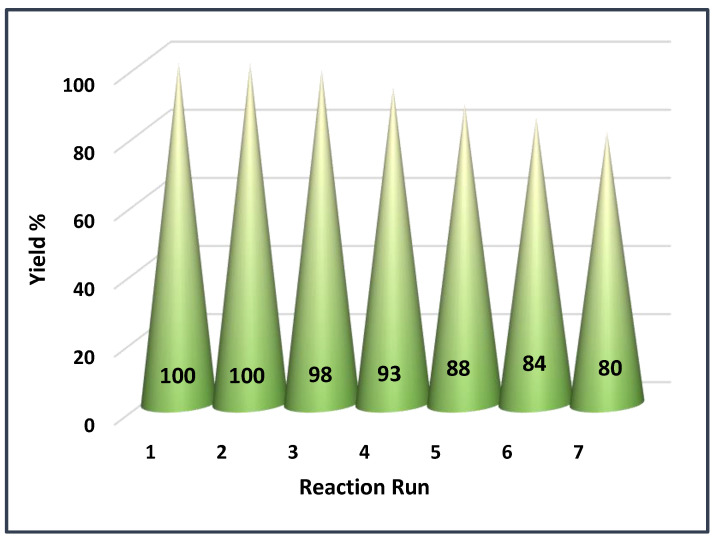
The recyclability of BC@GCN-P-IL for the model Biginelli reaction under its optimum reaction condition.

The XRD pattern of BC@GCN-P-IL was recorded, Figure S1, to study the crystal phase of the catalyst. According to the literature, the polymeric moiety on the BC@GCN is amorphous and it is expected to observe its characteristic band at 2θ = 20–30 ° [18]. The distinguishing band of BC@GCN is also appeared in the same range. As shown in Figure 7, in the XRD pattern of BC@GCN-P-IL a peak can be discerned in the range of 2θ = 25–30°. This result is in good accordance with the literature [18].

### 2.2. Catalyst Activity

In this research, we targeted preparation of a metal-free catalyst that could catalyze conventional chemical transformations in aqueous media under a mild reaction condition. In this regard, BC@GCN was prepared and then, a polymeric moiety that contained both IL and acid functionalities (–SO_3_H and –COOH) [66,68] was incorporated to the structure of the catalyst. It was assumed that both IL and acidic functionalities could effectively promote the acid-catalyzed reactions. To verify this assumption, first, the performance of BC@GCN-P-IL for Knoevenagel condensation that is a widely used chemical reaction was appraised. To this purpose, malononitrile and benzaldehyde were reacted at ambient temperature in water in the presence of 20 mg BC@GCN-P-IL. Gratifyingly, it was found out that after 2 h, the reaction completed and furnished the desired product in 100% yield. Next, it was elucidated whether reagents with different electronic features could also tolerate this reaction. In this line, several benzaldehydes with electron donating and electron withdrawing groups were examined as starting materials. Gratifyingly, the reactions of all of the studied substrates resulted in the corresponding products in excellent yields, Table 1. Even ketones and aromatic aldehydes could also undergo this reaction to give the products high yields.

To further reveal the merit of BC@GCN-P-IL for Knoevenagel condensation, the yield of the model Knoevenagel condensation under BC@GCN-P-IL catalysis was compared with some published methodologies. The yields of the desired product, recyclability and reaction parameters for each methodology are reported in Table 2. It is clear that this key reaction could be promoted by various catalysts, including metallic and non-metallic ones. In some tabulated procedures, use of precious metals or metal organic frameworks (MOFs) was reported that led to high yields of the desired product. Notably, fabrication of MOFs mostly needs precise procedure. On the other hand, use of non-metallic catalysts can be more environmentally benign. As shown, some non-metallic catalysts, such as glycine were not effective and resulted in moderate yields after a long reaction time. Regarding the reaction temperature, it can be seen that this reaction has been reported both at ambient and elevated temperatures. Regarding the reaction time, it can be concluded that the required reaction time for BC@GCN-P-IL was relatively low. Notably, in some tabulated procedure, the reaction times were shortened by use of ultrasonic irradiation or using metallic catalysts. Considering the recyclability of the catalyst, it can be seen that BC@GCN-P-IL exhibited good recyclability, comparable to some most of the tabulated catalysts.

The data presented in Table 2 could corroborate that metal-free BC@GCN-P-IL could perform under a mild reaction condition to give the condensation product in high yield.

Approving high activity of BC@GCN-P-IL for Knoevenagel condensation reaction, it was investigated whether this catalyst could promote more complex acid-catalyzed reactions. To this purpose, the one-pot, three-component Biginelli reaction for the synthesis of dihydropyrimidinones was targeted. Optimization of the reaction condition for a model Biginelli reaction, reaction of benzaldehyde, urea and ethyl acetoacetate, confirmed that using low content of the catalyst (0.03 g) in 1:1 mixture of H_2_O/EtOH at 50 °C, the reaction could proceed to furnish the desired product in 100% yield. Study of the generality of the present protocol, Table 3, also confirmed that this procedure could be generalized to various substrates with different electronic properties.

The results of the study of the catalytic activity of the catalyst confirmed that the developed catalyst could be considered as a versatile catalyst for promoting various acid-catalyst reactions.

### 2.3. Appraising the Contribution of Catalyst Moieties to the Catalysis

As BC@GCN-P-IL contained several functionalities, such as acidic and IL functionalities in its structure, the contribution of each moiety to the catalysis need to be studied. To shed light to this issue, preparation of some control samples and comparing their activities for a model reaction were imperative. To this purpose, the model Knoevenagel condensation reaction was selected for appraising the contribution of the catalyst moieties. For the first step, BC@GCN was prepared and used as a catalyst for promoting the model Knoevenagel condensation reaction. The result showed that using this catalyst the reaction led to only 60%, Table 4, of the desired product. Comparison of the activity of BC@GCN with that of BC@GCN-P-IL indicated superior activity of the latter. As the activity of BC@GCN was lower than that of BC@GCN-P-IL, it could be concluded that P-IL could contribute to the catalysis. To scrutinize the role of P-IL in the catalysis, two control samples, BC@GCN-P and BC@GCN-IL, were prepared. In BC@GCN-P, the polymeric network was formed from polymerization of AMPS and AA, while in BC@GCN-IL, the polymeric moiety was formed from polymerization of the as-prepared IL. In fact, in BC@GCN-P, the catalyst benefited from the acidic functionalities (–COOH and –SO_3_H), while in BC@GCN-IL, the catalyst possessed IL functionalities. Investigation of the catalytic activities of these two samples approved their superior activities compared to BC@GCN. This result established that the introduction of either IL or acidic functionalities was favorable for the catalytic activity. On the other hand, comparison of the activities of these two samples with that of BC@GCN-P-IL implied the higher activity of the latter. This issue corroborated that simultaneous incorporation of both IL and acidic functionalities in the backbone of the catalyst was beneficiary for the catalytic activity.

### 2.4. Recyclability of BC@GCN-P-IL

To commence the study of the recyclability of BC@GCN-P-IL, the recovered catalyst from the model Biginelli reaction was reused for the next run of this multicomponent reaction under the reported optimum reaction condition. The measurement of the yield of the model Biginelli reaction indicated that BC@GCN-P-IL maintained its activity for the second run of the reaction. Upon further reuse, slight decrease of the activity of BC@GCN-P-IL was discerned and the yield of the model Biginelli reaction reached to 80% after seven runs of the reaction, Figure 7.

**Figure 8:**
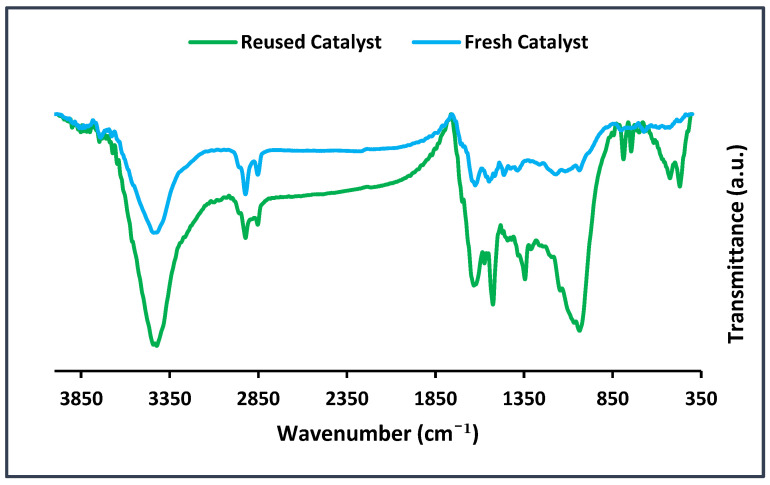
FTIR spectra of the recycled and fresh BC@GCN-P-IL after seven runs of the Biginelli reaction.

In the next part of this research, the recycled BC@GCN-P-IL was characterized via FTIR spectroscopy to elucidate the origin of the decrement of its activity. As shown in Figure 8, the FTIR spectrum of the recycled BC@GCN-P-IL was distinguished from fresh BC@GCN-P-IL. As shown, the intensities of the absorbance bands in fresh and reused catalysts were different. On the other hand, some new absorbance bands could be observed in the recycled catalyst. For example, the bands at 744–780 cm^−1^ and sharp bands at 1350–1400 cm^−1^ could be discerned in the FTIR spectrum of the reused catalyst. This issue can be attributed to the deposition of the products on BC@GCN-P-IL. In fact, coverage of the active sites of BC@GCN-P-IL could justify the observed loss of the activity.

## 3. Materials and Methods

In this research, *Zinnia grandiflora* petals were collected in the north of Iran and used as the biochar source. Other chemicals utilized for the preparation of the catalytic system included, 3-(trimethoxysilane) propyl methacrylate (3-TMSPMA), urea, 1-vinylimidazole (VIM), 1-bromobutane, 2,2′-azobis (2-methylpropionitrile) (AIBN), 2-acrylamido-2-methyl-1-propanesulfonic acid (AMPS), acrylic acid (AA), toluene, diethyl ether and EtOH. All of the so-called reagents were purchased from Sigma-Aldrich (Darmstadt, Germany). The chemicals used for performing the Knoevenagel condensation reaction and Biginelli reaction were aldehydes, malononitrile, ethyl acetoacetate, urea and EtOH, all provided from Sigma-Aldrich.

### 3.1. Apparatus

After preparation of the catalytic system, it was verified by various techniques. Fourier transform infrared (FTIR) spectral analysis was performed with KBr pellets method via PERKIN-ELMER-Spectrum 65 (Bruker, Germany). Thermogravimetric analysis (TGA) was accomplished using a (METTLER TOLEDO, model Leicester, Leicester, UK) under N_2_ atmosphere with heating rate of 10 °C min^−1^. The X-ray diffraction (XRD) pattern was recorded on Siemens, D5000 apparatus using graphite monochromatic Cu-Kα (Karlsruhe, Germany). Field emission scanning electron microscope (FE-SEM) images and energy dispersive spectroscopy (EDS) were gathered by using MIRA 3-XMU (Tescan Co., Brno, Czech Republic). All of the organic compounds synthesized from Biginelli and Knoevenagel condensation reactions were synthetic. Hence, their characterization was performed by comparing their spectral data (^1^H NMR, ^13^C NMR and FTIR) with authentic samples.

### 3.2. Synthesis of the Catalyst

#### 3.2.1. Synthesis of BC@GCN

Preparation of biochar-based graphitic carbon nitride was carried out according to the previously reported procedure with some modification [75,76]. In this work, *Zinnia grandiflora* petals, collected from the north of Iran, were utilized for the first time as biosources. Biosource was rinsed with deionized water repeatedly, air-dried and then transformed to powder by using a mixer. In order to prepare biochar-based graphitic carbon nitride, urea (5 g) was thoroughly mixed with *Zinnia grandiflora* powder (5 g) and ground for 10 min in a mortar. The resulting powder was then calcined in a tubular furnace under N_2_ atmosphere at 450 °C for 2 h. Afterwards, the furnace was cooled down to room temperature and the product, BC@GCN, was taken out.

#### 3.2.2. Synthesis of BC@GCN-V

To vinyl-functionalize BC@GCN, 2 g of BC@GCN was dispersed in toluene (60 mL) under stirring for about 30 min. Then, 2 mL of 3-TMSPMA was gradually added to the abovementioned suspension. After stirring for 10 min, the mixture was refluxed for 24 h. Finally, the solid product was filtered, washed with toluene and dried at 60 °C overnight.

#### 3.2.3. Synthesis of BC@GCN-P-IL

First, IL monomer was prepared. In this regard, VIM (2 mL) and excess amount of 1-bromobutane were mixed in a 50 mL round-bottomed flask and kept at 70 °C for 24 h under stirring. Upon completion of the reaction, the obtained mixture was allowed to cool at ambient temperature, and then diethyl ether was introduced to furnish a light-brown viscous IL. Finally, the diethyl ether was removed from the reaction system under vacuum and the product was washed with MeOH for three times and dried at room temperature.

In the next step, using the as prepared IL monomer and two other monomers (AA and AMPS), BC@GCN was adorned with a polymeric moiety, P-IL, containing IL and acidic functionalities. Typically, BC@GCN-V (1 g) was added to EtOH/H_2_O (30 mL, 2:1) and suspended by ultrasonic. Afterward, AIBN (0.1 g) as the polymerization initiator was introduced to the so-called suspension under inert atmosphere and the obtained mixture was heated up to 70 °C. After 30 min, aqueous solutions of AMPS (1 mL), AA (1 mL) and IL (1.5 g) were added into the above mentioned mixture and the polymerization was continued for one day under stirring conditions. Finally, the solid product was filtered, washed tree times with EtOH and dried under vacuum at 60 °C.

### 3.3. Evaluation of the Catalytic Activity

#### 3.3.1. Knoevenagel Condensation Reaction

Typically, all of the reagents, i.e., aldehyde (1 mmol) and malononitrile (1.2 mmol) were mixed in H_2_O and then BC@GCN-P-IL (0.02 g) was added. The obtained mixture was stirred for 2 h at ambient temperature. Knoevenagel condensation was simply traced by TLC (Merck Co., Readington, NJ, USA). At the end, MeOH (10 mL) was added and BC@GCN-P-IL was separated through conventional filtration. The recovered catalyst was reused five times in the model reaction after washing with MeOH and drying at 70 °C overnight. To obtain the Knoevenagel condensation products, the solvent was evaporated under vacuum and products were purified by column chromatography (ethyl acetate/hexane 1:5) (Agilent, Santa Clara, CA, USA). All of the products were synthetic [66] and their characterization was performed by comparing their melting points and spectral data (^1^H NMR and ^13^C NMR) with authentic samples. Moreover, the yields of the reactions were estimated by using GC (Agilent, USA).

#### 3.3.2. Biginelli Reaction

This one-pot reaction was performed by mixing aldehyde (1 mmol), ethyl acetoacetate (1 mmol), urea (1.3 mmol) and BC@GCN-P-IL (0.03 g) in H_2_O: EtOH (10 mL, 1:2) and heating up to 50 °C. Monitoring the reaction by TLC confirmed that the reaction completed after 3 h. After completion of the reaction, MeOH was poured to the reaction vessel and BC@GCN-P-IL was separated, washed with EtOH and reused seven times in model reactions without a significant loss of activity. To obtain DHPMs, the solvent in the filtrate was evaporated and the resulting DHPM was rinsed with EtOH. Furthermore, with the aid of column chromatography (using ethyl acetate/hexane mixture) the product was purified. All of the products were synthetic [77] and their characterization was performed by comparing their melting points and spectral data (^1^H NMR and FTIR) with authentic samples. Moreover, the yields of the reactions were estimated by using GC.

## 4. Conclusions

Using *Zinnia grandiflora* petals and urea as a nitrogen source, a novel biochar-based graphitic carbon nitride, BC@GCN, was fabricated. To introduce functionalities on BC@GCN, it was vinyl functionalized and reacted with AMPS, AA and IL in the presence of AIBN. The formed polymeric network on BC@GCN contained both acidic (–COOH and –SO_3_H) and IL functionalities and rendered the hybrid system, BC@GCN-P-IL, an efficient catalyst for promoting acid catalyzed reactions such as Knoevenagel condensation and Biginelli reaction in aqueous media under a very mild reaction condition. The developed protocols could be generalized to various substrates. Moreover, BC@GCN-P-IL could be successfully recovered and reused for several reaction runs. Using some control catalysts, it was found that introduction of P-IL that contained the acidic and IL functionalities on BC@GCN dramatically increased the catalytic activity.

## Figures and Tables

**Table 1:**
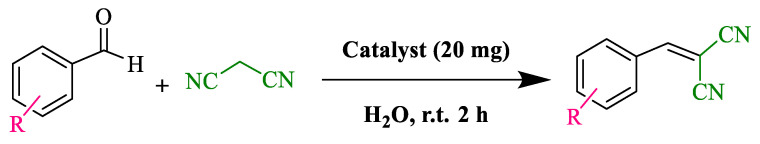
Knoevenagel condensation reactions of various aldehydes with malononitrile catalyzed by BC@GCN-P-IL ^a^.

NO.	Substrate	Yield (%) ^b^
1	Benzaldehyde	100
2	4-NO_2_-benzaldehyde	98
3	2-NO_2_-benzaldehyde	95
4	3-NO_2_-benzaldehyde	95
5	4-Me-benzaldehyde	98
6	4-MeO-benzaldehyde	97
7	2-MeO-benzaldehyde	95
8	4-Cl-benzaldehyde	100
9	Furfural	90
10	Acetophenone	85

^a^ Reaction condition: Substrate (1 mmol), malononitrile (1.2 mmol) and BC@GCN-P-IL (20 mg) in H_2_O at 25 °C in 2 h. ^b^ Isolated yields.

**Table 2:** The comparison of BC@GCN-P-IL-based procedure for the model Knoevenagel condensation reaction (malononitrile and benzaldehyde) with some reported ones [69,70,71,72,73,74].

Entry	Catalyst	Temp. °C	Solvent	Time (h:min)	Recycle Run	Yield (%)	Ref.
**1**	Glycine	25	[6-mim]PF_6_	22:00	2	77	[69]
**2**	Caffein-SiO_2_@Fe_3_O_4_	60	H_2_O (ultrasonic irradiation)	00:06	5	94	[70]
**3**	Activated Hf-UiO-66-N_2_H_3_	25	EtOH	4:00	5	98	[71]
**4**	[H_3_N^+^-CH_2_-CH_2_-OH][CH_3_COO^−^]IL	25	Solvent free	<1:00	5	90.9	[72]
**5**	PdNi@GO	25	H_2_O/EtOH	00:08	5	95	[73]
**6 ^a^**	[Zn_2_(TCA)(BIB)_2,5_]·(NO_3_)	60	Solvent free	1:00	4	>99	[74]
**7**	BC@GCN-P-IL	25	H_2_O	2:00	5	100	This work

^a^ [Zn_2_(tricarboxytriphenyl amine)(1,3(imidazol-1-ylmethyl)benzene)_2,5_].(NO_3_).

**Table 3:**
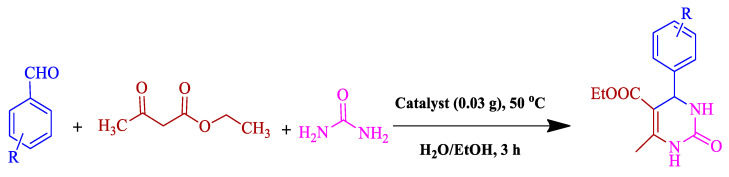
BC@GCN-P-IL catalyzed synthesis of dihydropyrimidinones ^a^.

NO.	Substrate	Yield (%) ^b^
**1**	Benzaldehyde	100
**2**	4-NO_2_-benzaldehyde	98
**3**	2-NO_2_-benzaldehyde	90
**4**	3-NO_2_-benzaldehyde	93
**5**	4-Me-benzaldehyde	95
**6**	4-MeO-benzaldehyde	96
**7**	2-MeO-benzaldehyde	90
**8**	4-Cl-benzaldehyde	95

^a^ Reaction condition: substrate (1 mmol), ethyl acetoacetate (1 mmol), urea (1.3 mmol), catalyst (30 mg), H_2_O/EtOH (10 mL) at 50 °C in 3 h. ^b^ Isolated yields.

**Table 4:** The comparison of the efficiency of BC@GCN-P-IL with control samples for model Knoevenagel condensation reaction (reaction of malononitrile and benzaldehyde) under optimum condition.

Entry	Catalyst	Yield %
**1**	BC@GCN	60
**2**	BC@GCN-P	85
**3**	BC@GCN-IL	90
**4**	BC@GCN-P-IL	100

## Supplementary Materials

FE-SEM image of BC@GCN is available online.
