# Supplementary material for: Biochar-Based Graphitic Carbon Nitride Adorned with Ionic Liquid Containing Acidic Polymer: A Versatile, Non-Metallic Catalyst for Acid Catalyzed Reaction
Source: Molecules. 2020 Dec 16;25(24):5958. doi: 10.3390/molecules25245958 (PMC7766038; doi:10.3390/molecules25245958)
Supplement: Supplementary file 1 [file molecules-25-05958-s001.pdf]

Supporting information

Biochar-based graphitic carbon nitride adorned with ionic liquid containing acidic polymer: a versatile, non-metallic catalyst for acid catalyzed reaction

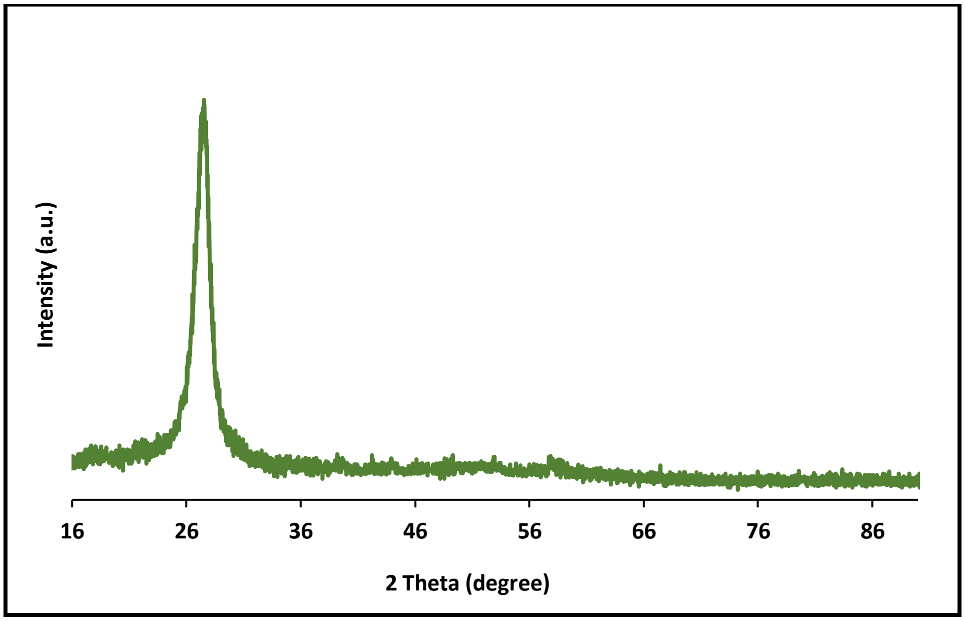

Figure S1. XRD pattern of BC@GCN-P-IL.

^{1}HNMR spectra for selected Knoevenagel condensation products

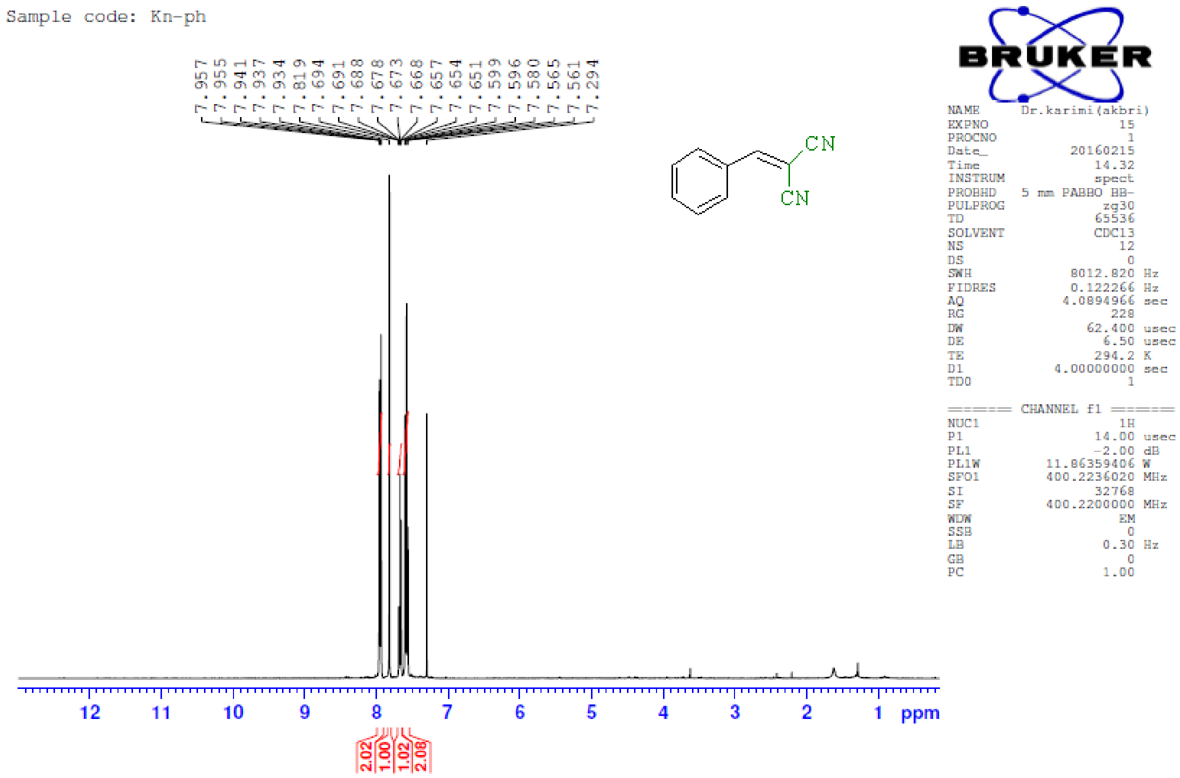

Figure S2. ^{1}HNMR spectrum of Knoevenagel condensation reaction of malononitrile and benzaldehyde.

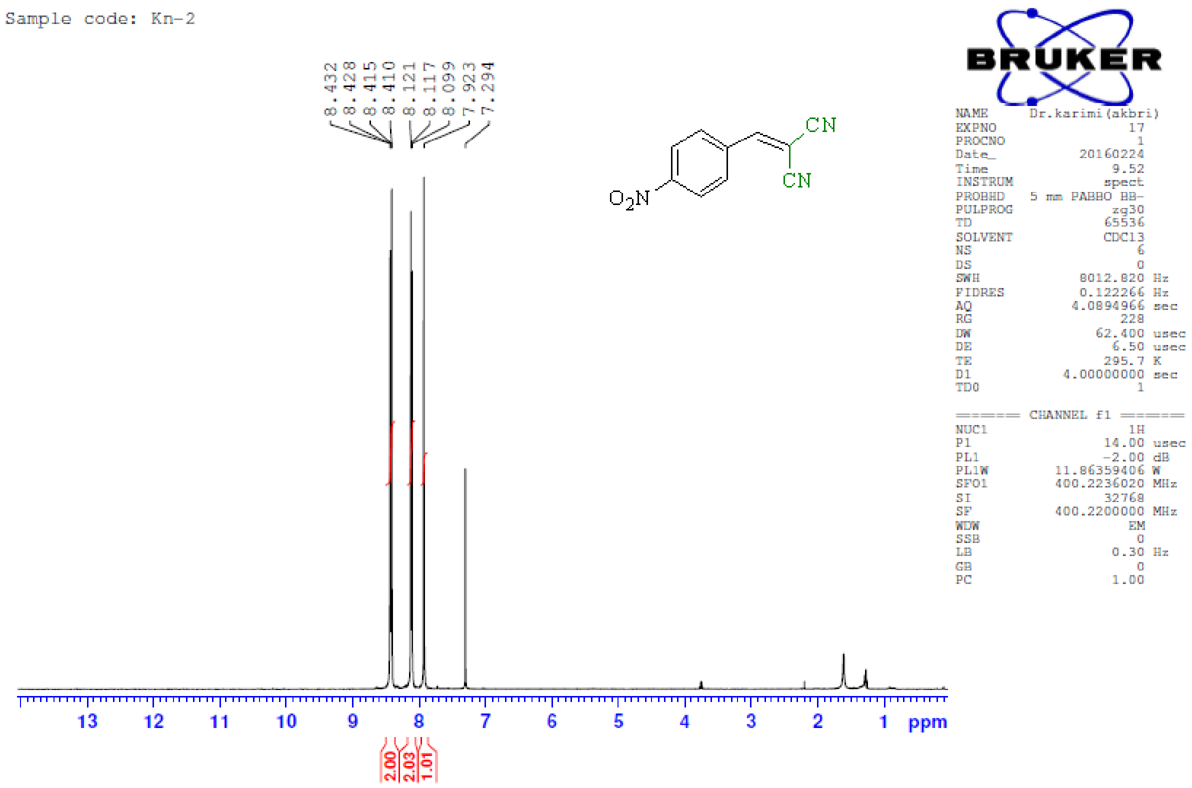

Figure S3. ^{1}HNMR spectrum of Knoevenagel condensation reaction of malononitrile and 4-nitrobenzaldehyde.

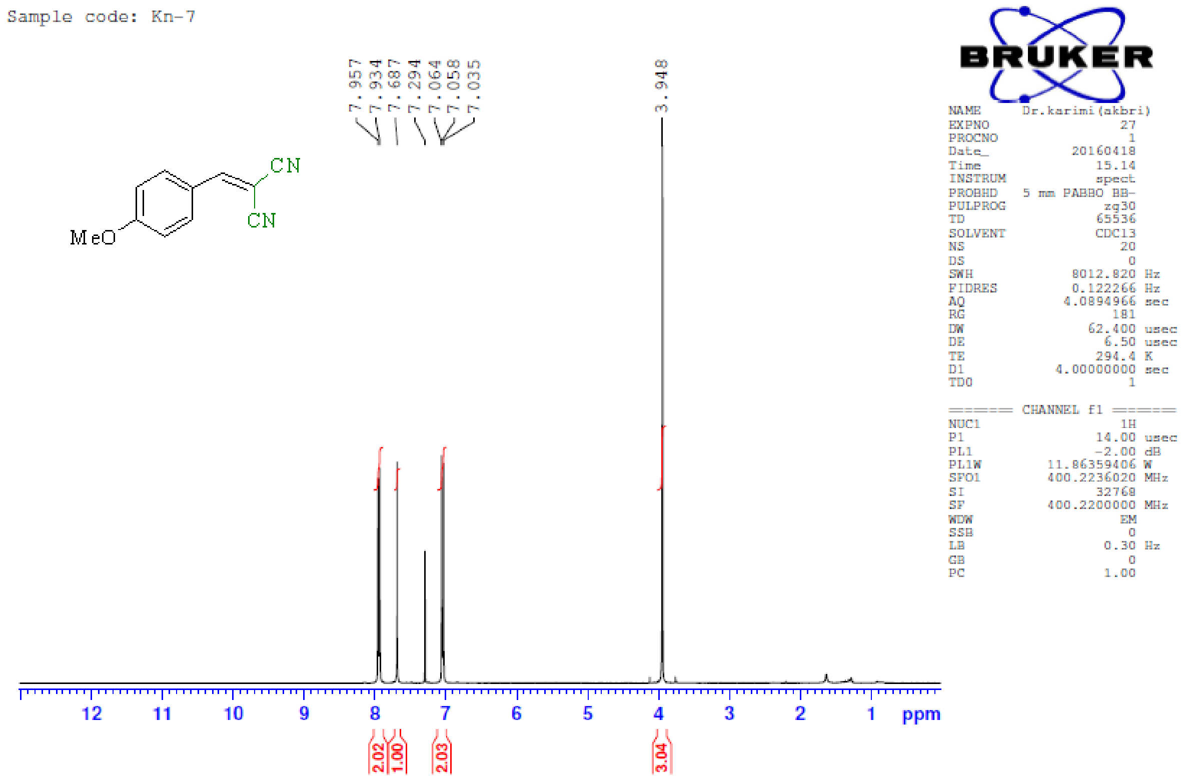

Figure S4. ^{1}HNMR spectrum of Knoevenagel condensation reaction of malononitrile and 4-methoxybenzaldehyde.

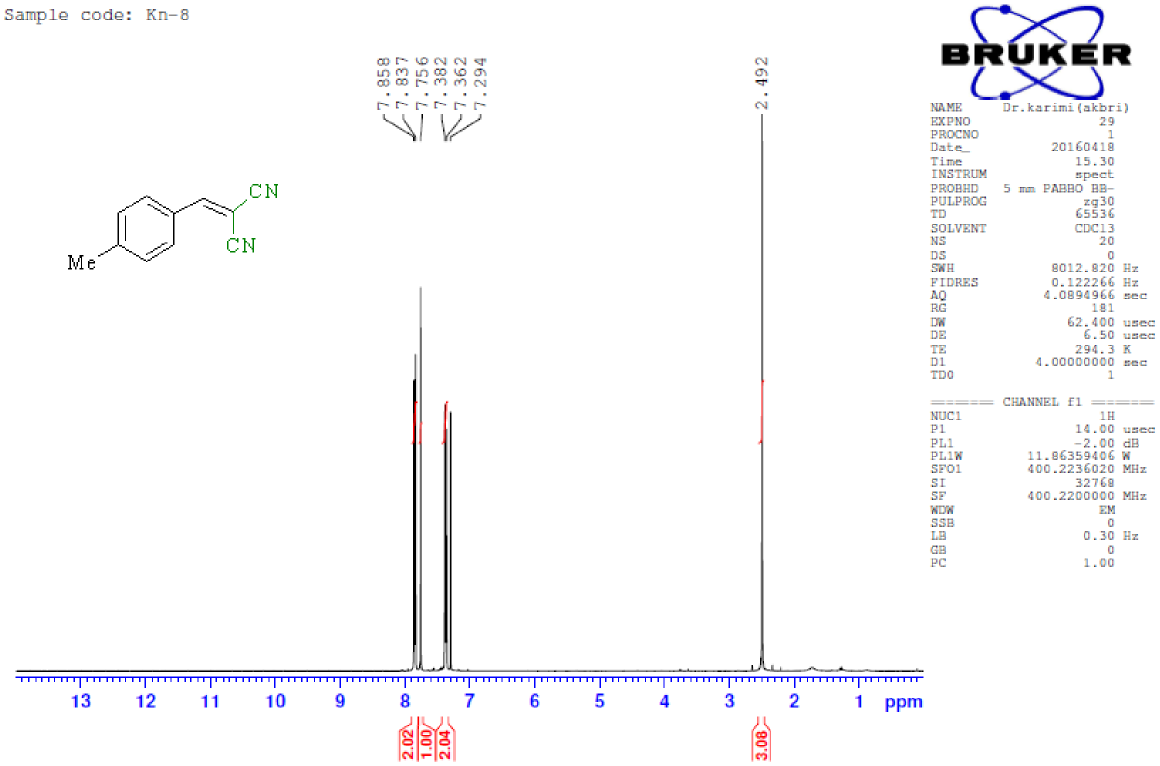

Figure S5. ^{1}HNMR spectrum of Knoevenagel condensation reaction of malononitrile and 4-methylbenzaldehyde.

^{13}CNMR spectra for selected Knoevenagel condensation products

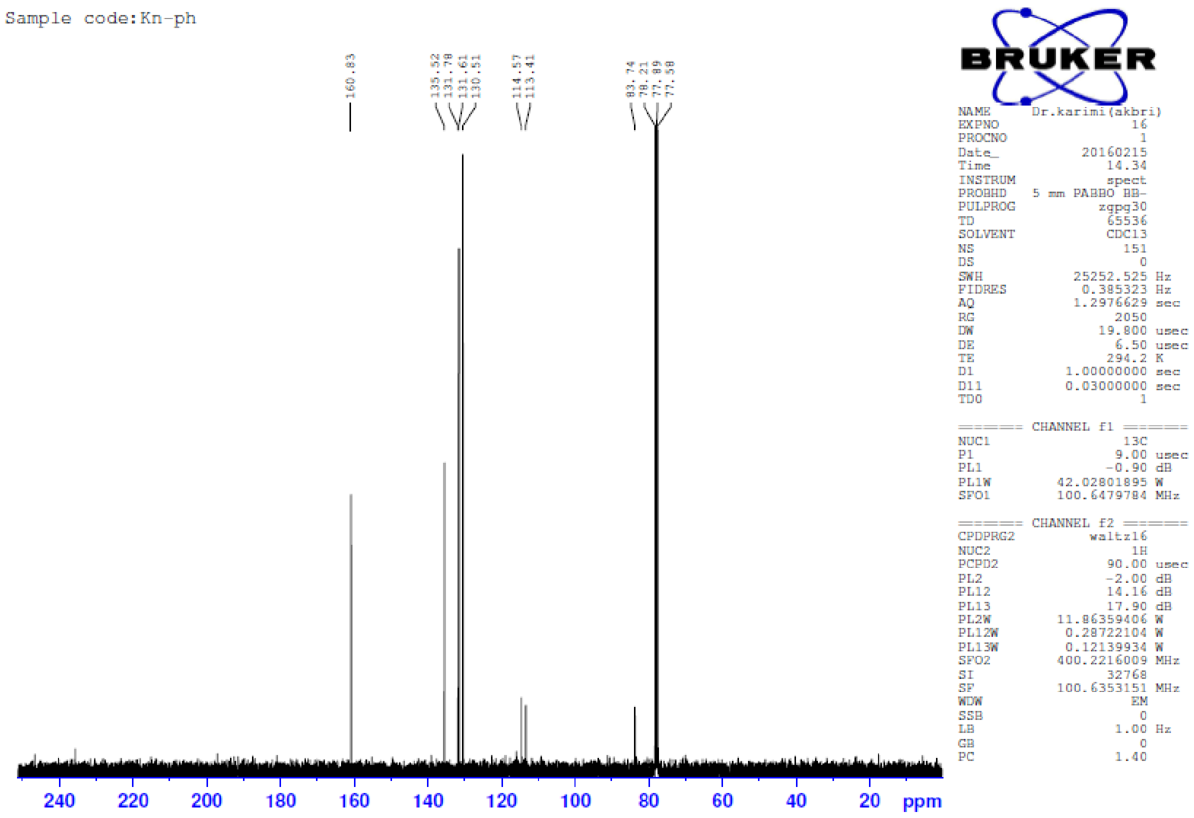

Figure S6. ^{13}CNMR spectrum of Knoevenagel condensation reaction of malononitrile and benzaldehyde

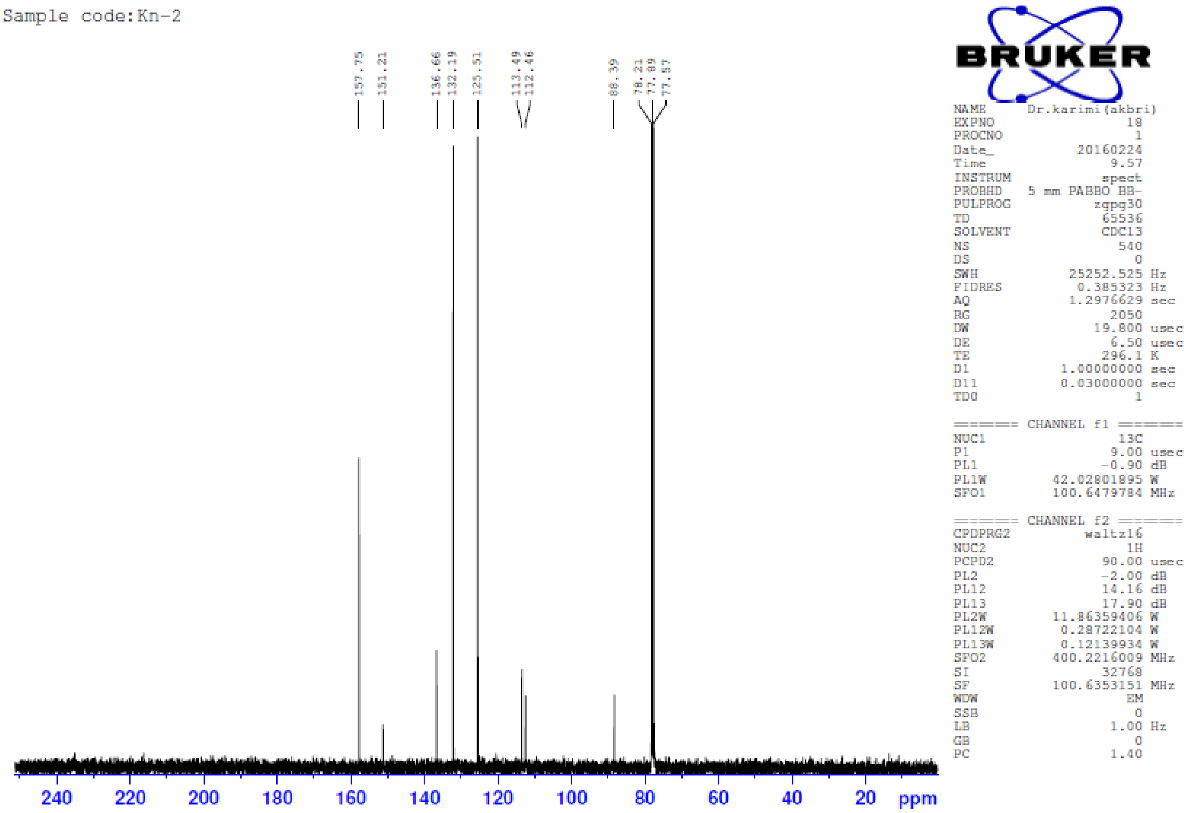

Figure S7. ^{13}CNMR spectrum of Knoevenagel condensation reaction of malononitrile and 4-nitrobenzaldehyde.

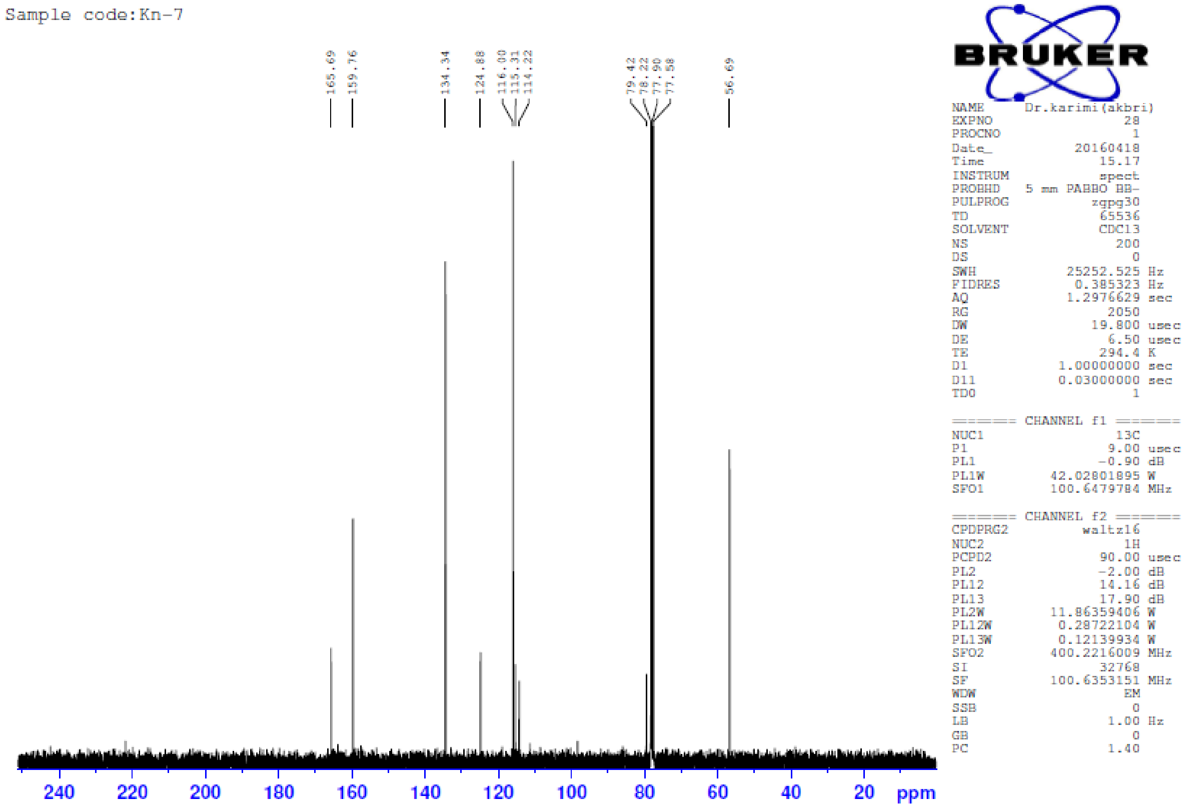

Figure S8. ^{13}CNMR spectrum of Knoevenagel condensation reaction of malononitrile and 4-methoxybenzaldehyde.

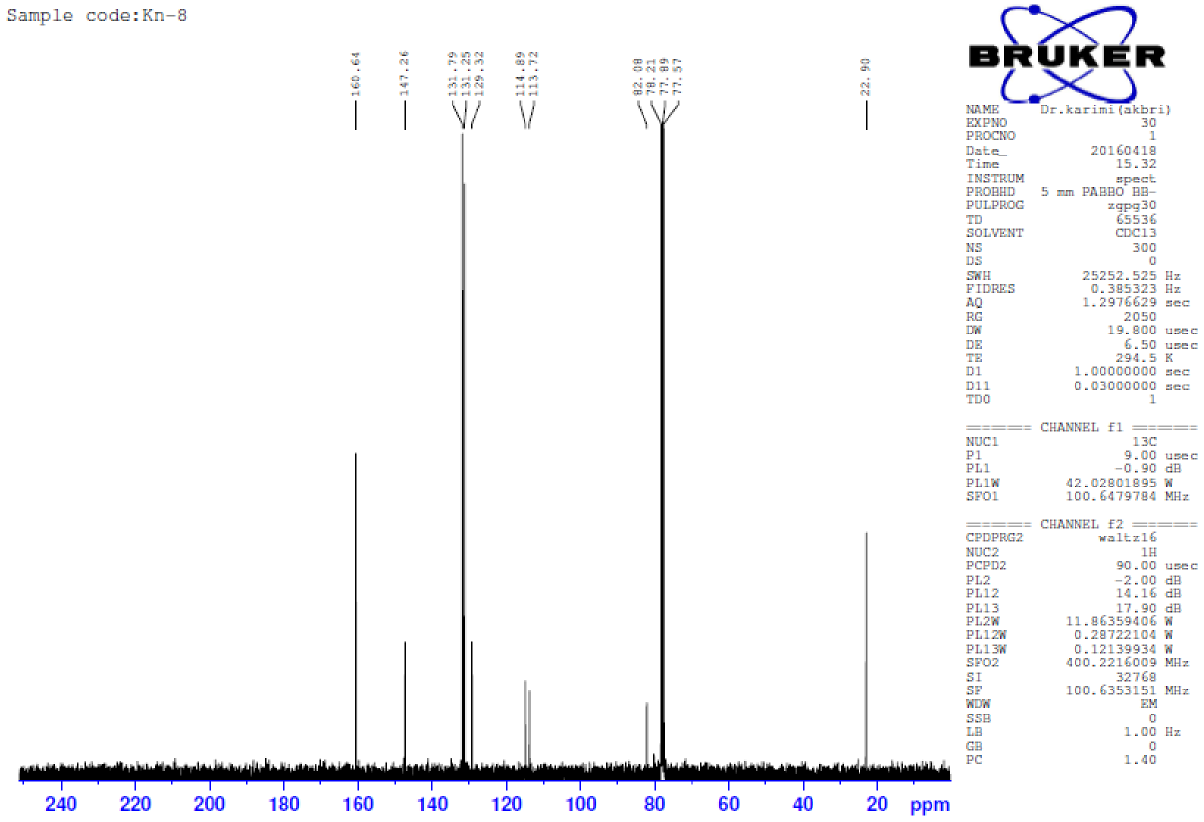

Figure S9. ^{13}CNMR spectrum of Knoevenagel condensation reaction of malononitrile and 4-methylbenzaldehyde.

^{1}HNMR spectra for selected dihydropyrimidinones

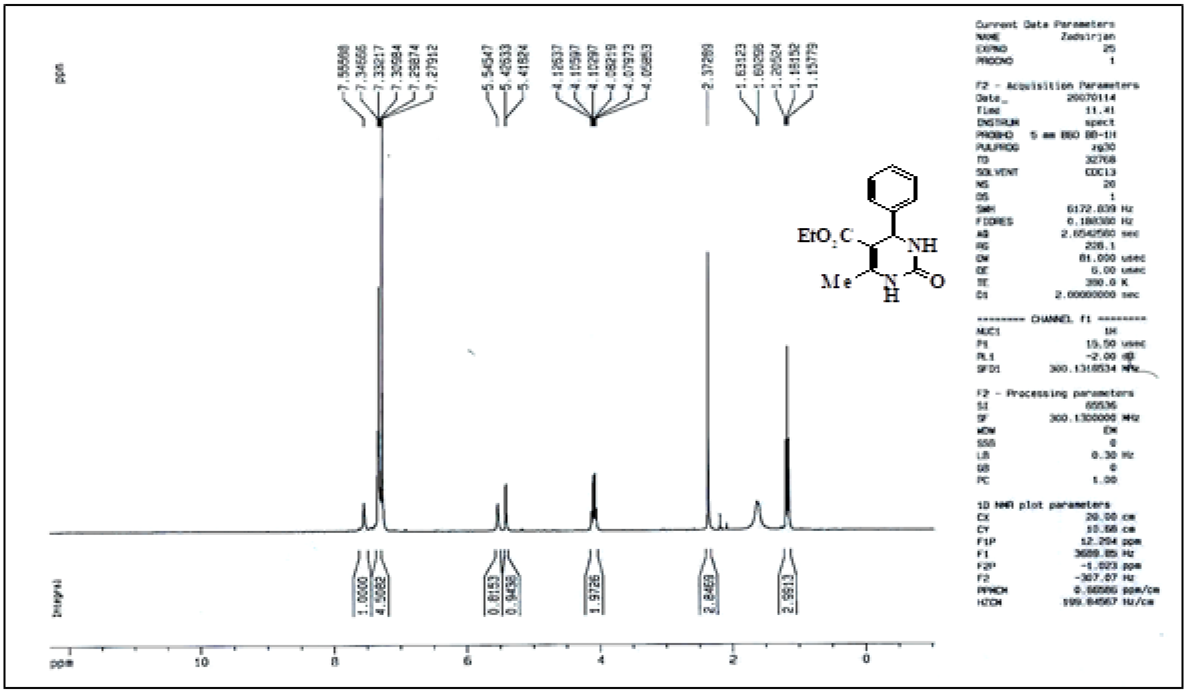

Figure S10. ^{1}HNMR spectrum of dihydropyrimidinone synthesized from reaction of urea, ethyl acetoacetate and benzaldehyde.

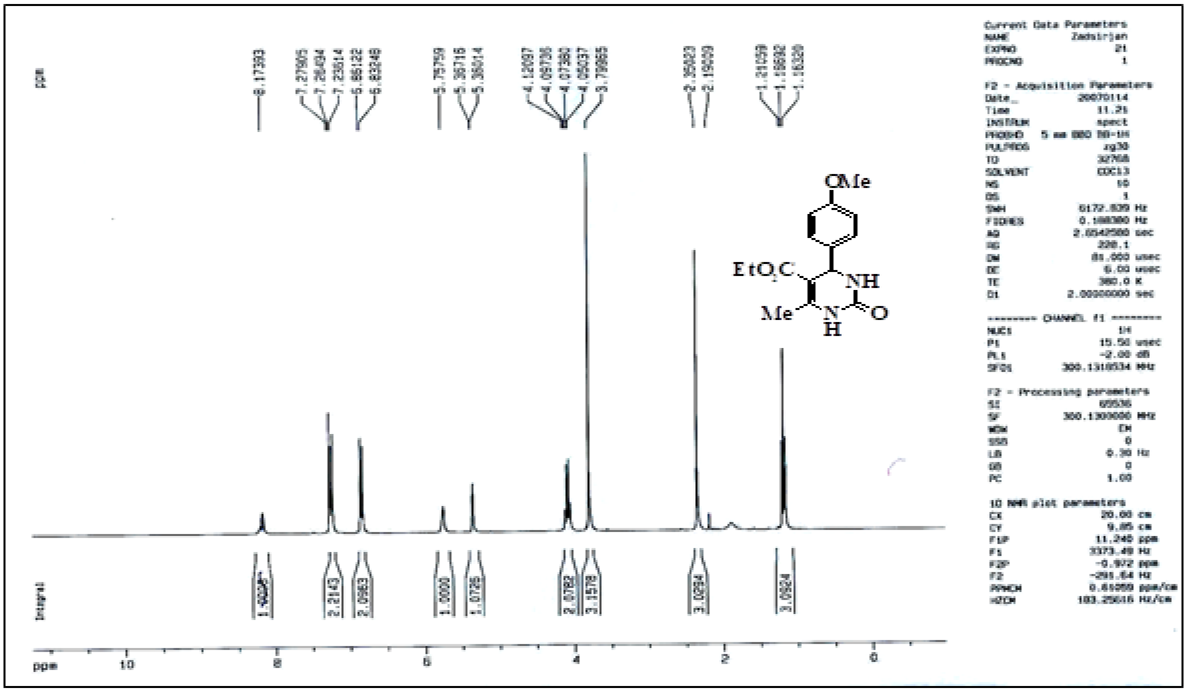

Figure S11. ^{1}HNMR spectrum of dihydropyrimidinone synthesized from reaction of urea, ethyl acetoacetate and 4-methoxybenzaldehyde.

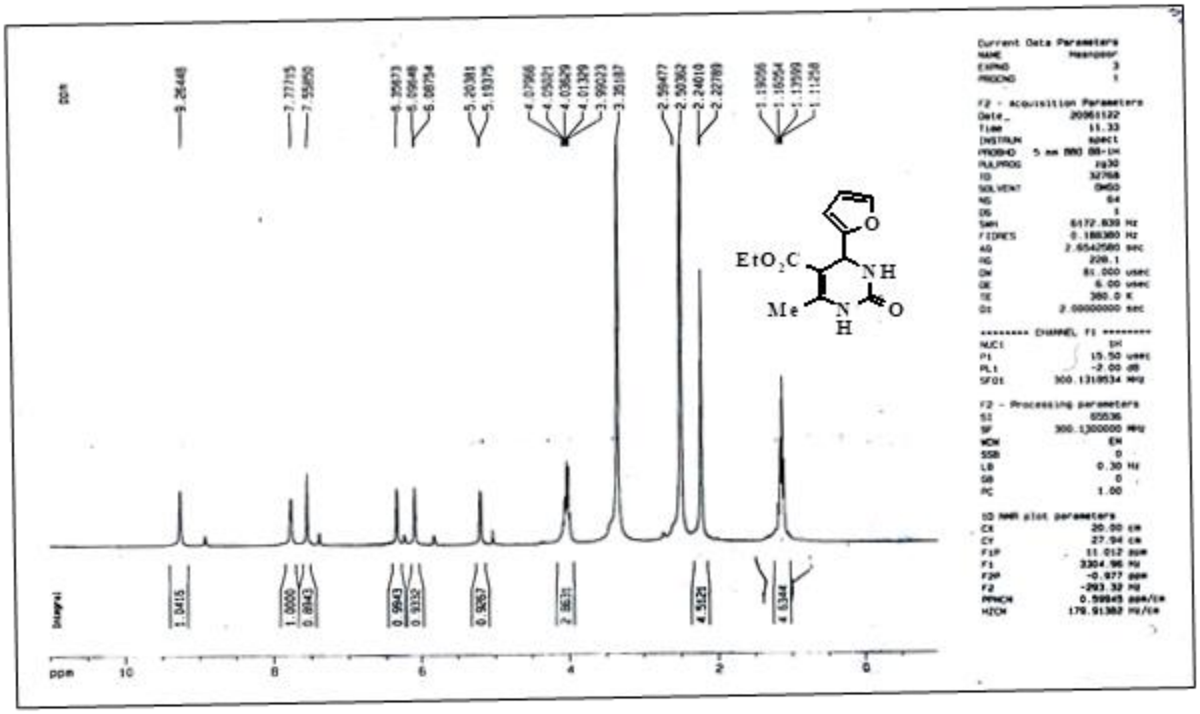

Figure S12. ^{1}HNMR spectrum of dihydropyrimidinone synthesized from reaction of urea, ethyl acetoacetate and furfural.

FTIR spectra for selected dihydropyrimidinones

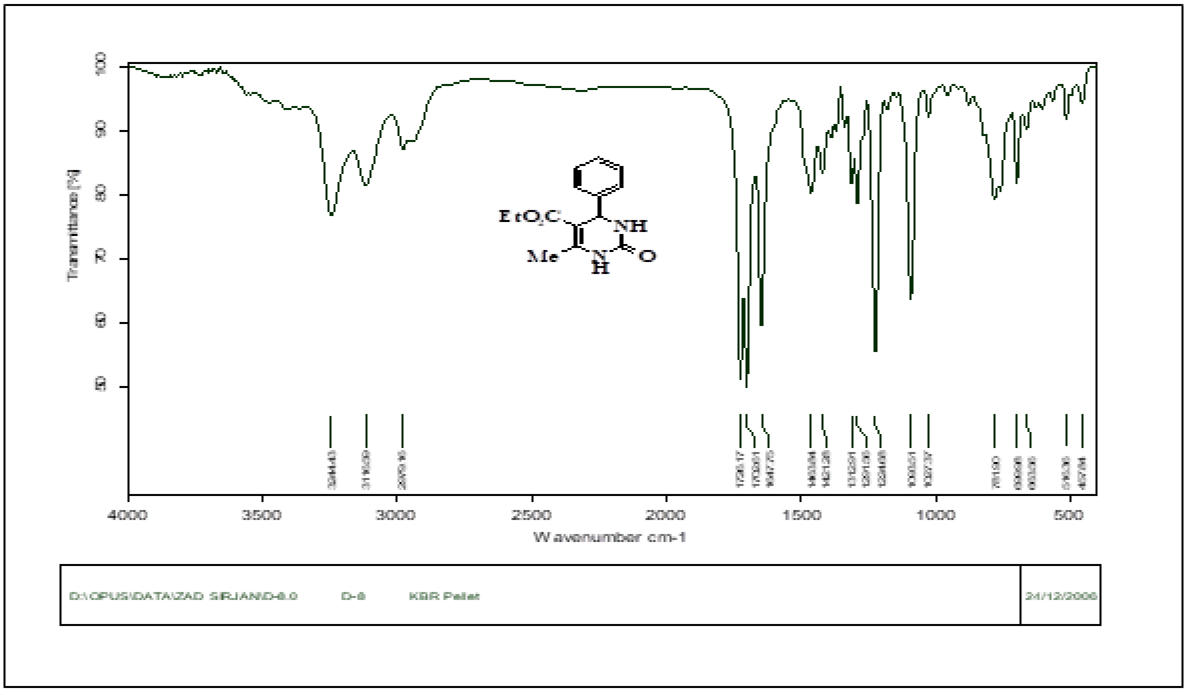

Figure S13. FTIR spectrum of dihydropyrimidinone synthesized from reaction of urea, ethyl acetoacetate and benzaldehyde.

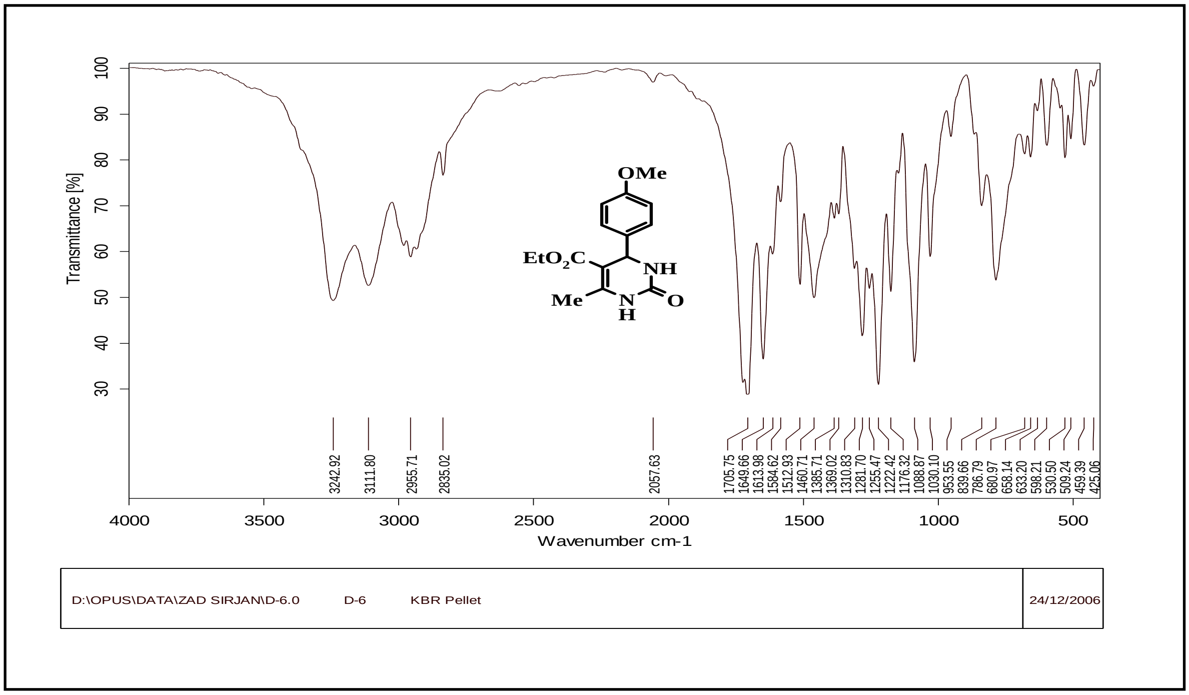

Figure S14. FTIR spectrum of dihydropyrimidinone synthesized from reaction of urea, ethyl acetoacetate and 4-methoxybenzaldehyde.

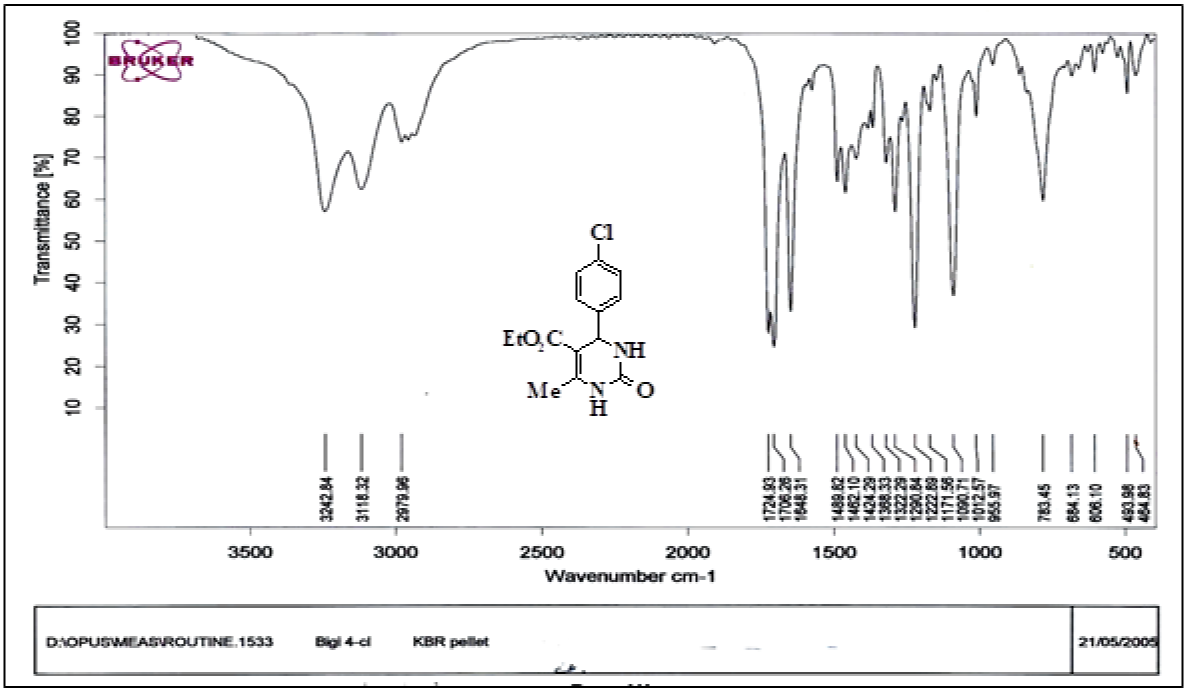

Figure S15. FTIR spectrum of dihydropyrimidinone synthesized from reaction of urea, ethyl acetoacetate and 4-chlorobenzaldehyde.

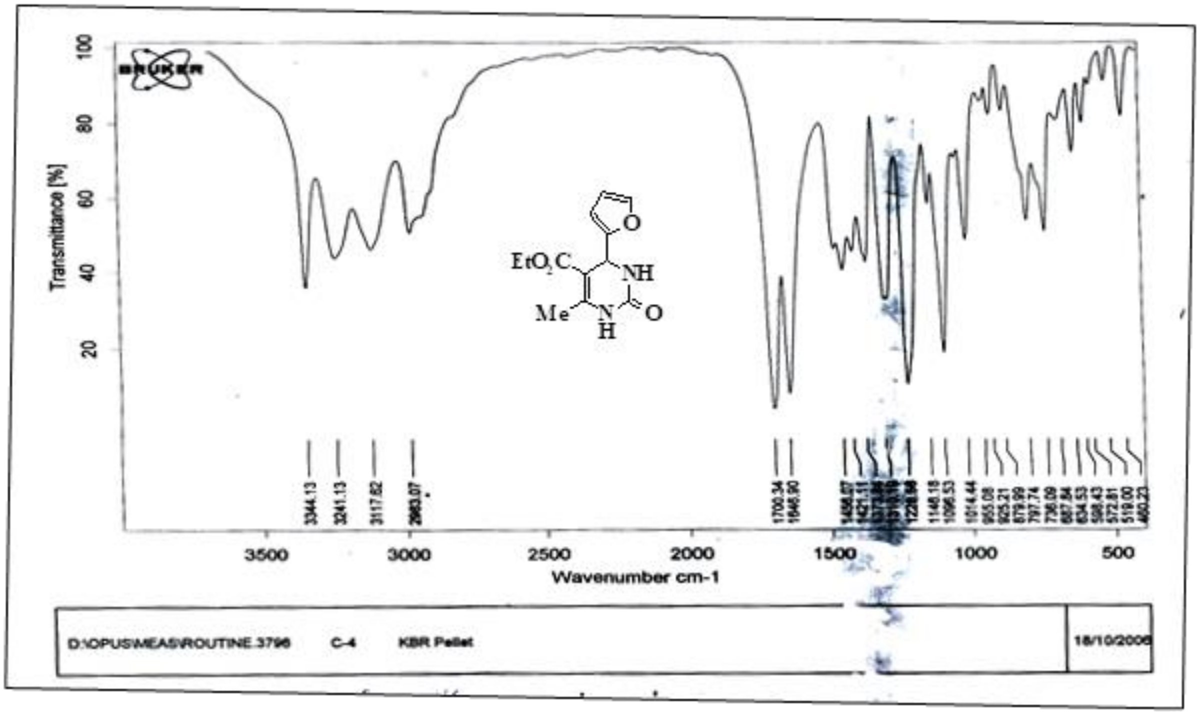

Figure S16. FTIR spectrum of dihydropyrimidinone synthesized from reaction of urea, ethyl acetoacetate and furfural.
